# Rural eHealth Nutrition Education for Limited-Income Families: An Iterative and User-Centered Design Approach

**DOI:** 10.2196/jmir.1148

**Published:** 2009-06-22

**Authors:** Nancy L Atkinson, Sandra L Saperstein, Sharon M Desmond, Robert S Gold, Amy S Billing, Jing Tian

**Affiliations:** ^1^Department of Public and Community Health Public Health Informatics Research LaboratorySchool of Public HealthUniversity of MarylandCollege ParkMDUSA; ^2^School of Public HealthOffice of the DeanUniversity of MarylandCollege ParkMDUSA; ^3^Center for Substance Abuse ResearchUniversity of MarylandCollege ParkMDUSA; ^4^University of Maryland School of MedicineBaltimoreMDUSA

**Keywords:** Qualitative research, website design, rural health, nutrition management, exercise, obesity

## Abstract

**Background:**

Adult women living in rural areas have high rates of obesity. Although rural populations have been deemed hard to reach, Internet-based programming is becoming a viable strategy as rural Internet access increases. However, when people are able to get online, they may not find information designed for them and their needs, especially harder to reach populations. This results in a “content gap” for many users.

**Objective:**

User-centered design is a methodology that can be used to create appropriate online materials. This research was conducted to apply a user-centered approach to the design and development of a health promotion website for low-income mothers living in rural Maryland.

**Methods:**

Three iterative rounds of concept testing were conducted to (1) identify the name and content needs of the site and assess concerns about registering on a health-related website; (2) determine the tone and look of the website and confirm content and functionality; and (3) determine usability and acceptability. The first two rounds involved focus group and small group discussions, and the third round involved usability testing with individual women as they used the prototype system.

**Results:**

The formative research revealed that women with limited incomes were enthusiastic about a website providing nutrition and physical activity information targeted to their incomes and tailored to their personal goals and needs. Other priority content areas identified were budgeting, local resources and information, and content that could be used with their children. Women were able to use the prototype system effectively.

**Conclusions:**

This research demonstrated that user-centered design strategies can help close the “content gap” for at-risk audiences.

## Introduction

Obesity is a national priority health issue [1], and the problem is particularly severe among rural populations, with the highest rate of obesity in adult women living in rural areas [2]. People living in rural areas experience several nutrition-related health disparities, including heart disease and diabetes [3]. Compounding this situation is the high rate of poverty among rural residents [4]. Rural populations have been deemed hard to reach with general communication methods [5] and with technology-based media [6].

More recently, Internet-based programming has been used to reach difficult-to-reach populations, including those in rural areas [7]. The purpose of this study was to conduct formative research to design and develop an Internet-based health education intervention promoting nutrition and physical activity among rural mothers with limited resources using an iterative user-centered approach.

### Internet Access

In the past, rural populations have been shown to have lower rates of Internet use [8]. Recently, however, rural populations have had rates of Internet use similar to those of people living in other geographic locations [9]. Concerns have shifted to focus on whether rural Internet users will be susceptible to a new access barrier, having low-speed, dial-up connections rather than broadband [9]. Despite this concern, the rate of broadband adoption among rural Americans has been increasing. Between 2007 and 2008, broadband rates increased from 31% to 38%, an increase of 23% in one year [10].

Rural populations have different barriers to access than the general public. The main reason people in the general public do not go online is lack of interest [9], but most rural, limited-income mothers who were not yet online intended to use the Internet in the future [11]. The main barrier preventing use has been the expense of the hardware and software.

Rural populations have similar reasons as others for wanting to use the Internet, including searching for health information. Among Internet users in 2006, four out of five adults reported using the Internet to locate health information [12]. A recent study with rural, limited-income mothers (n = 146) also found that a large majority of those who used the Internet (86%) reported searching for medical information online, and two thirds reported viewing health-related websites [11]. Similarly, people living in rural areas have demonstrated no differences in their online searches for Medicaid and Medicare compared to people living in urban areas [14].

### Content Divide

Access to the Internet is only part of the digital divide. Once individuals get online, information and tools they want and need may not be available [13]. More than 50 million Americans cannot find or use needed online materials and services [13]. Even if materials are available, they are often complex and require advanced literacy skills [15]. This “content gap” leaves the promise of the Internet unfulfilled for many, including those with low incomes, low literacy, limited English, and disabilities [13]. This gap affects online health information and tools, which have been found to have, on average, a tenth grade reading level [16]. Therefore, recommendations have been made to improve the reach of health websites by working to meet the needs of underserved populations [13].

### User-Centered Design

Given increases in access and high rates of interest in Internet use in general and for health promotion specifically, technology-based interventions offer a potential means to reach rural populations [11]. With proper design and dissemination, eHealth programs could be a critical tool in the elimination of health disparities [16]. An important methodology to create appropriate online content is user-centered design [17]. In user-centered design, the target audience is involved in all stages of the development process in order to create a website that best meets users’ needs [17]. According to the evidence-based guidelines, “The current research suggests that the best way to begin the construction of a Web site is to have many different people propose design solutions (i.e., parallel design), and then to follow up using an iterative design approach” [17].

Some of the more promising Internet-based interventions include tailored communication, which is a strategy that can improve the relevance and appeal of health messages [18]. However, tailored communication is based on demographic and other personal information provided by the individual. Technology has been implicated in various privacy issues because of its role in facilitating the gathering, aggregating, and disseminating of information [19]. The development of Internet-delivered interventions must therefore assess the audience’s concerns about trust and privacy [15,20]. Most Internet users (84%) are concerned about others gaining access to their personal information, and about half (54%) are concerned about getting online medical information from unqualified sources [20]. Despite these concerns, over half (54%) have given personal information so that they can use a specific website, and another tenth report that they would provide personal information under certain circumstances [20]. Privacy may be less of a concern for those individuals who are actively engaged in seeking and sharing health information [21]. When asked the three most important ways that digital communication has changed how they share or receive health information, health-engaged individuals identified having access to more up-to-date health information (42%), access to new information (40%), and more immediate access to information (38%); however, only 10% felt that digital health communication made them more concerned about the privacy of their health information [21]. Understanding the kinds of information people would share is important in the development of a website providing tailored health information.

### Purpose

The purpose of this study was to conduct three rounds of a user-centered design process to guide the development of a website to support and extend the goals of the Food Stamp Nutrition Education Program (FSNEP) in Maryland. The priority audience of this online program was mothers with limited incomes living in the state’s rural counties because of their key role in guiding nutrition and health choices for their families. The iterative process was designed to answer the following research questions:

- Round 1: (1) How acceptable is the idea of the proposed website to the priority population? (2) What are the preferences for the proposed website name and content? (3) How will limited-income mothers react to the idea of providing information about themselves during registration and log-in procedures proposed for the website?
- Round 2: (1) What design components will be most appealing and understandable? (2) What content and features would the priority audience want and expect in a website about nutrition, physical activity, and food budgeting?
- Round 3: (1) How acceptable is the prototype website? (2) Is the prototype website easy to navigate and use?

## Methods

### Sample

The priority population for the concept testing was limited-income adult females (age 18 or older and having an income < 185% of the federal poverty level) living in five counties in Maryland. Another selection criterion was having at least one child enrolled in school (preschool to eighth grade) in order to obtain feedback on making health choices in the context of a family. If potential participants were not currently receiving food stamps, eligibility for the concept and message testing was based on household income and household size.

### Recruitment

Recruitment was conducted using multiple methods. Flyers were posted in key locations and distributed by community service providers (eg, Department of Social Services personnel, extension educators) to their eligible clients. In the second round, faith-based leaders also assisted in distributing flyers. Updated lists of food stamp recipients were obtained from the Maryland Department of Human Resources to recruit persons directly via telephone. Reminder calls were made to registered participants prior to the focus groups and interviews in an effort to increase attendance. In the first two rounds, a free meal was offered as an incentive. No incentives were offered in the third round; however, the participants in one county were given the opportunity to sign up for free Internet accounts, which may have provided some incentive.

### Instrumentation

The focus groups and interviews were conducted with structured guides that built upon the findings of each previous round as the intervention was drafted and developed. See Table 1 for the topics and questions covered in the three rounds. Building upon the needs assessment findings of the previous year, the main purposes of the first round were to test the overall concept of the website, its name, and the idea of having people register. Based on these findings, three conceptual designs and a draft content outline were developed, and the interview guide was developed to be consistent with the designs and content. In the second round, participants were asked to evaluate the potential designs for the home page, identify which design they preferred, and recommend what content areas the site should include. Based on the second round, a functional prototype was developed with the recommended subsections and draft content pages.

In the third round of testing, individual interviews and usability testing protocols were used to assess acceptability and ease of use. The interviewee was asked to explore the website using a mouse as an interviewer observed and asked questions about the website. The interview began with general questions about the home page and purpose of the site. Next, home page features were described and pointed out to the interviewee, who was then asked to choose which features to look at in greater detail. Allowing the user to click on the features by order of interest allowed us to assess which features were the most interesting and compelling while getting more specific information about each feature. We also observed how well users were able to navigate back and forth between the features and the home page. This strategy was also used to explore the secondary pages—Feed Your Mind, Cooking Class, Stay Connected, Activities—in that a brief tour was given, then the user was able to pick which pages to visit and explore while answering questions about each. The ability to navigate between secondary content areas and their features was also observed to determine how well users could find information within the program. The interview ended with overall questions about website acceptability and suggestions for how to improve it.

The interview guides were developed with input from the Maryland Cooperative Extension. All three protocols were submitted to the Institutional Review Board at the University of Maryland and received approval prior to the initiation of each phase of the study.

**Table 1:** User-centered research questions by round of research process

Round	Section	Illustrative Questions
Round 1	Introduction	Who [here] has ever used the Internet? We want to create a website for low-income families with information to address food and physical activity needs. *Reaction to mock website (see Figure 1)* What are your feelings and thoughts about this idea?
	Input on Name	What are your ideas about what to name a website with this kind of information? If you wanted to find information about healthy eating or how to exercise for yourself or for your kids, what phrase or words would you enter into the computer? *Reactions to four draft concepts*
	Input on Registering	Has anyone [here] ever been on a website and had to create a log-in name and password? How would you feel about having to type in your name and password into a website each time you use it? How would you feel about registering on a website so you could get information on your specific interests and needs? What kind of personal information would you be most/least comfortable sharing when registering on a website? *Reactions to 27 types of information*
Round 2	Introduction	How many people here have ever used the Internet? What are some of your favorite websites? What do you like about these sites? Dislike?
	Reactions to Three Draft Websites	*Show three different examples of the website, one at a time (see Appendix 1)* What is the first thing that strikes your eye about this website? What do you like/dislike about this website? Is there anything confusing about this website? Who do you think this website is for? What do you think about the design of this website? How could we improve this website?
	Reactions to Proposed Content: Reading Room Cooking Class Community Center Tool Box	When you hear the name of this area, what is the first thing that comes to your mind? What kind of information would you expect to see here? If you visited this menu, which of these choices interest you the most/least? Overall, which one of these areas would interest you most/least?
Round 3	Introduction	Have you used the Internet before?
	Responding to Pilot Website Home Page	*Show the home page of the website.* What do you think the purpose of the website is? What is your reaction to how it looks?
	Responding to Home Page Features	*Provide brief tour of weekly poll, tip of the day, suggestion box, and ask the coach.* Which of these do you want to look at first? How well do you think this section accomplishes its purpose? Do you like or dislike it? Why? *Repeat with other three home page items in order of interest.*How could it be improved?
	Responding to Content	*Provide them with a brief tour/overview of each area (Feed Your Mind, Cooking Class, Stay Connected, Activities).* Which of these do you want to look at first? What do you think of this section? Do you like or dislike it? Why? How could it be improved? *Repeat with other three content areas in order of interest.*
	General Website Review	What did you like about this website? What was your favorite part? What did you dislike about this website? Is there anything confusing about this website? How could we improve this website? Would you recommend the website to others?

### Data Collection

#### Round 1

Focus groups (n = 5) were held in five counties in February and March 2004. Groups ranged in size from 1-9, for a total of 28 participants. A trained moderator led all focus groups, which lasted approximately 90 minutes and were audiotaped; a second staff member took notes.

#### Round 2

Round 2 focus groups (n = 3) were held in May and June 2004 in three counties that would be piloting the website. Due to low recruitment rates in one county, three individual interviews were also conducted at an adult literacy program center in addition to the focus group. There were 4-5 participants in each group and three interviews, for a total of 16 participants. A moderator and note taker conducted the 90-minute groups, which were also audiotaped.

#### Round 3

Individual interviews were conducted in each of the three intervention counties. Researchers staffed several common areas where low-income mothers frequented, for approximately 7 hours at a time. These included sites such as an adult learning center and residential community center. Participants were asked to attend at a time that was convenient for them for a period of approximately 30-45 minutes. A trained moderator led all eight interviews, and, when possible, a second staff member took notes on a structured participant observation review form to capture both comments and actions as the individual moved through the pilot website.

### Data Analysis

Data from the focus groups and interviews were analyzed using note-based analysis. This technique involves analyzing the notes taken during the sessions and any summary notes made by the moderator and note-takers immediately after the session, with the audiotapes used as needed for verification of findings [22]. The notes were analyzed to identify key issues and common themes by question and by area of the website. A grid was constructed to provide an overview summarizing the content of the discussions. Multiple reviewers (n = 3) were used to verify the emergent themes and issues.

## Results

### Round 1: Reaction to Proposed Intervention, Names, and Registration

**Figure 1:**
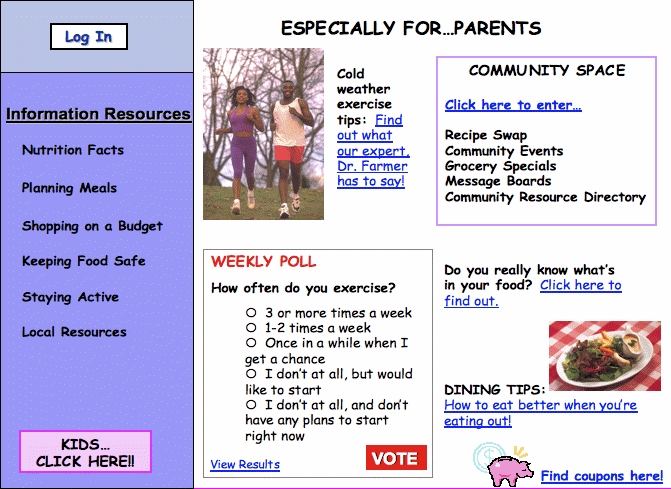
Mock website home page

When asked whether they had ever used the Internet, 22 of 28 participants reported that they were either current or past Internet users. When presented with a mock home page with proposed website content (Figure 1), overall reactions were positive.

Participants were interested in the website, especially the “community space” area that would allow them to access local information about their communities. Participants also expressed interest in content related to food budgeting, or “smart shopping,” and nutrition and physical activity information related to their children. They wanted content tailored to limited-income families that would take their monetary resources into account when providing budgeting information and recipe ideas. They provided several suggestions for topics to include in the website:

- Nutrition: How to pack a healthy meal; Cooking for picky eaters; Nutritional values of different foods; Interactive activities and tools (ie, nutrition assessments); Meal planning, budgeting, shopping on a budget, coupons

- Physical Activity: How to track exercise levels for kids and adults; Ideas for staying active, particularly in-home exercise options; Ways to stay motivated for exercise; Cooking; Weekly recipes that use low-cost, healthy ingredients; Ways to use different kinds of foods in recipes; Nutrition facts in recipes

- Content for Children: Healthy snack ideas for children; Cooking with children; How to deal with childhood obesity; Age-specific information about helping children be active; Printable coloring pages and age-specific games

- Local Resources: Message boards for community groups/events; Nutrition resources that provide low-cost food items; Low-cost recreational options, especially during the cold seasons

Participants were asked to react to four potential website names. The names were ranked in the following order: (1) Eat Smart, Be Fit; (2) Families Fit for Life; (3) HealthPath – The Path to Healthy Living; and (4) Healthy Community, Healthy Family, Healthy Me. The vast majority preferred the name “Eat Smart, Be Fit,” describing it as “catchy” and “straight to the point.” This name would give the user a good sense of what would be presented on the website and would appeal to families, particularly women and children, and to people who wanted to diet, eat healthfully, or exercise. Those who disliked it felt that the name was not representative of all the information on the website. The other names were less preferable because they did not give the user a clear, comprehensive idea of what the website was about. The last two suggested names listed above were considered long, confusing, and hard to remember.

With respect to registration, most participants had previously logged in to websites and were familiar with this practice. Remembering names and passwords was considered difficult for some participants. Some were concerned about the security of personal information entered during the log-in procedure. Others were not concerned, finding the process of logging in to a website to be routine and enjoying the personalized content they received. They preferred not to have to do it each time they accessed a website.

Each group was presented with 27 different types of information that could be gathered in a registration process. Groups were willing to provide information about health, nutrition, and physical activity goals and practices in order to get tailored advice. However, several items were considered sensitive: personal contact information (address, phone, email), household information (number of children in household, number of adults in household, income), personal health information (health problems, weight), and demographics (employment status, education level, food stamp status). See Table 2 with findings by topic.

**Table 2:** Comfort with giving personal information during website registration

Log-In Item	Proportion Comfortable	Favorable Findings	Unfavorable Findings
First/last name	4/5	Used to giving this information out	Don’t want to give last name
Gender	4/5		
Street address	2/5	Acceptable if site is secure	
Zip code	5/5		
Telephone number	5/5		Make optional
Email address	4/5	Interested in receiving a newsletter	Make optional
Age	5/5	Use age ranges	
Number of children in household	3/5		Too personal
Number of adults in household	3/5		
Topics of interest	5/5		
Health status	4/5		Some uncomfortable
Health goals	5/5		
Personal health problems	4/5		Should not ask about sensitive health problems
Family health problems	5/5		
Eating habits	5/5		
Food buying habits	5/5		
Food budgeting habits	5/5		
Exercise habits	5/5		
Computer habits	4/5		Unnecessary
Internet habits	4/5		Too personal
Height	4/5		Make optional
Weight	2/5	Use weight ranges	Sensitive
Employment status	3/5	Use categories; kind of personal but okay	Make optional
Education level	4/5		Unnecessary, make optional
Household income	4/5	Use ranges	Not relevant
Food stamp status	4/5		Unnecessary, determine from income and household size
How heard about website	5/5		

### Round 2: Design and Content Preferences

When presented with three different websites (see Multimedia Appendix), participants identified what they liked and disliked about each. With respect to graphics and pictures, participants recommended that photographs include people representing a mix of ethnic backgrounds, body types, and ages. People should be shown in active poses and wearing comfortable, but not sloppy, exercise clothes. They also liked graphics that helped explain website content. In particular, they liked the graphic on the first website that depicted a large family having a barbecue. “Everybody can join in,” one person said. Others noted that the children in the picture were “probably talking about food” and that the picture showed children “being taught what’s good for them and what’s not.” The first website also used vegetables to create graphics in the header, and they appreciated how this linked the site to nutrition.

Participants liked the layout of the second website, saying that having the links divided by boxes made it easier to see what was on the page and where to click. All favored the idea of having drop-down menus rather than menus that required them to click to the next page before being able to view the submenus. They also expressed interest in the use of colorful graphics for website links. The third website was considered inappropriate and boring, mainly for its photo (“looks like a homeowner’s page”) and color scheme. The prominent display of the acronym FSNEP (Food Stamp Nutrition Education Program) was confusing because most participants were unfamiliar with it. Finally, the menu buttons on this website design were unclear.

Participants were asked to respond to the individual menu items and related content, and they appeared somewhat confused regarding the names of the menu options. They recommended changing the names of several menus to promote both clarity and interest among users. Participants preferred names that were “fun and catchy,” that were inclusive of many users, and that would help the user anticipate the content.

The proposed content areas were received positively (Table 3). Participants seemed most interested in ensuring that content would appeal to a broad audience, including children. Participants offered suggestions for content, focusing on materials that could be used to ease a mother’s daily tasks, such as planning meals on a budget. They wanted access to information on how to make healthier meals and be more active. Both information and interactive tools were of interest to the priority audience to help them accomplish these goals. They also stated an interest in local resources that could help them save money on food and provide low-cost exercise options.

**Table 3:** Reactions to and recommended new content for website sections during Round 2 focus groups and interviews

Section of Website	Reactions	Recommendations for New Content
Reading Room	Expected material pertaining to nutrition and exercise; Concerned about the amount of reading that might be involved; Wanted a name that was fun and catchy	Planning meals and menus; How to get kids to eat nutritiously; Eating on a budget; Assistance with counting calories; Ways to exercise and stay active; Links to other websites
Cooking Class	Was the area of greatest interest; Thought this section would be of interest to children; Thought people who do not like to cook would not be interested	Healthy recipes; Cooking with children; Menus for children; Proper kitchen skills; Low-fat cooking techniques
Community Center	Expected information about activities in the community; Confused initially about what kind of information the “Ask the Expert” feature would provide	Local activities and events; Grocery specials; Community centers; Recreational facilities; Access to legal and medical advice
Tool Box	Thought the name made the purpose of the section unclear; Expected to find information to help them use the website; Overweight people less interested in using a body mass index (BMI) calculator	Exercise and activity logs; Food journal and calorie counter; Quizzes

Participants were next asked to review sample messages representing website content to assess their appropriateness. These messages were previously determined through readability testing to be at a sixth grade or lower reading level. Participants were asked to use their own words to describe the meaning of the paragraph and what they learned from it. They were able to read and understand these messages with relative ease, and they were enthusiastic about the content they reviewed. As a result, we concluded that a sixth grade or lower reading level was appropriate.

### Round 3: Acceptability and Ease of Use

Two of the eight participants in Round 3 had no Internet experience; however, even these respondents required very little direction on how to use a mouse and navigate the pages. When asked their impression of the intended purpose of the website, participants thought the purpose was to promote healthier eating and cooking habits, an interest in one’s health, and spending their food stamps or money wisely on products that would further a healthier lifestyle. They also felt that the site was trying to get people to engage in physical activity. They thought the pictures suggested a family-oriented site, promoting togetherness and healthy eating habits.

Participants liked the visual appearance of the home page and found it “eye catching,” colorful, and easy to use and understand. Suggestions for improving the home page included posting photos of diverse family configurations, including single-parent families, and people exercising and grocery shopping. Also, several participants felt that more colors and graphics should be added.

Next, participants reviewed and reacted to the prototype content and materials in each section (see Table 4 for their specific suggestions). Among the home page features, half the participants selected to view the tip of the day first; the “Ask the Coach” feature ranked second in interest. In terms of the content areas, users appeared most interested in areas that related to raising children and cooking with children. They were interested in the interactive features so that they could be more proactive in terms of meal planning, accessing local resources, and getting motivated to manage their weight. Of least interest were the suggestion box feature on the home page and the section on food safety in the cooking area.

**Table 4:** Suggestions for improvement of website sections/features during Round 3 interviews

Section/Feature	Suggestions for Improvement
**General Website**	Add brighter pictures and color to the background. Add humor to the content. Highlight key information into bullets, so text is less dense. Increase font size to make it easier to read. Simplify text, or provide access to a dictionary of terms. Make links more prominent. Provide more content targeted toward children.
**Content Areas**	
Planning Meals	Provide sample meals plans and meal planner tools.
Eating on a Budget	Add information on the food groups and recommended amounts. Provide food budgeting recommendations and worksheets.
Raising Healthy Kids	Add topics (eg, staying fit and eating right during and after pregnancy). Include links to local parks and recreation areas.
Keeping Food Safe	Add information on importance of using paper versus cloth towels. Provide information on the health effects of food additives.
Keep It in Season	Remove the section on canning.
Healthy Cooking	Add more information on healthy cooking. Provide nutrition information for people with illnesses (eg, diabetes).
Cooking with Kids	Provide lunch and recipe ideas for kids. Provide brown bag lunch ideas that won’t spoil.
**Interactive Features**	
Tip of the Day	Place in the box a link to related information about the tip. Provide ideas on implementing the tip in the context of a busy lifestyle.
Ask the Coach	Be clear that users can ask questions. Provide background information on “coaches.” Give time estimate for posting answers. Provide more visual aids, graphics next to questions and answers.
Community Events	Give instructions for expanding events on the calendar into full view. Provide wider range and greater number of events. Create a form letting community members post events on the website.
Grocery Specials	Provide recipes that can be used to shop for food items. Provide information on how to select foods at the grocery store. Create a grocery list builder or printable form to plan grocery lists. Create a shopping game.
Community Directory	Rename the food resources section to clarify the content found there. Add resources (eg, free/low-cost exercise classes, community pools).
My Activity Log	Stress the importance of checking with a physician before exercising. Provide field for recording specific upper and lower body exercises. List options for types of exercise. Give guidelines for weekly exercise and exercise intensity. Create online logging so that you don’t have to print out a log.
My Food Log	Provide daily caloric, fat, fiber, etc. guidelines. Provide information on servings (serving size, number of servings). Provide information on the importance of eating regular meals.
My Pledge	Include a timeline/calendar function to plan health behavior changes.
Bean Game	Use a more appealing graphic than a bean for the game, such as apples. Provide feedback telling the person how well they did at the game.

When asked to identify their favorite areas, participants generally selected the interactive components, such as Time Management, Ask the Coach, and the Activity and Food Logs. A few participants preferred the content areas, including Staying Active, Cooking Class, and Feed Your Mind. When asked what they disliked, participants identified specific components, such as individual graphics and tips, rather than large sections or areas of the website. For example, one person wanted to change the bean graphic to an apple graphic in the bean game because people might not like to eat beans. Another person recommended removing information on canning.

Overall, participants felt that the website would be helpful to them, they would like using it, and they would recommend it to others. Many wanted more information on the website that could be used with children, and several wanted to see a specific children’s area.

While reviewing the website, users were asked to comment on how easy the website was to use. In particular, participants noted that the menu system was easy to navigate. Other website features on the home page, the Suggestion Box and Ask the Coach, were found to be easy to use. Some participants offered specific suggestions to help improve user interaction with the site (see Table 3). Some participants stated that they were visual learners and preferred more graphics to text. These participants felt too much reading was required and would have preferred more activities. Many participants thought the font size throughout the site was too small to read comfortably. They also suggested reducing the density of the text on some pages and using more bulleted text to make the content easier to read.

## Discussion

The concept development process enabled the project to evolve through iterative review and comment by the intended end users. The discussion describes how the findings from each round built upon each other and previous research.

### Round 1: Proposed Website

The first series of focus groups allowed us to gain insight into the experiences of limited-income women in using the Internet and provided significant direction related to website naming and content development. Most importantly, participants’ strong and enthusiastic interest in the project confirmed that the idea of the proposed website was acceptable to and welcomed by limited-income women.

The name “Eat Smart, Be Fit” was favored by an overwhelming majority and was therefore selected as the brand name for the website and related project materials. Participants also liked the idea of receiving practical suggestions about food and fitness that were consistent with their income and location of residence. This finding is consistent with previous research [23] that found that people with limited incomes living in rural communities want practical local information, such as neighborhood events and local service agencies. Information that would allow them to better care for their children also resonated with the participants.

Similar to previous research [20], this study found that participants would provide certain personal information during a website registration process—such as health, nutrition, and physical activity goals and practices—in order to receive tailored advice. While some were willing to provide sensitive information, they wanted these types of questions to be optional rather than required. Because the participants had concerns that some items were irrelevant or unnecessary, any information requested in a registration procedure should also be justified, and an explanation about the purpose of gathering this information should be provided to potential registrants.

### Round 2: Concept Testing

The second round of testing revealed that the content, organization, and overall design, or “look and feel,” of the website strongly influenced whether users liked the website or not. Graphical images that supported the content and images that featured people who looked like them and their families resonated better with participants. They wanted a colorful website with graphics of diverse families engaging in physical activity or preparing and/or consuming healthy foods. This finding enabled the website design to be focused in a way to better convey the intended messages, and it was consistent with the usability guideline to use images that work for the users rather than the designers [17].

Round 2 also built upon the findings of the first round by confirming that the content chosen for further development was of interest: healthy meals for the family, eating on a budget, and local resources. However, it also demonstrated that the content needed to be adjusted to better fit the audience’s needs and expectations, such as changing menu labels to be more appealing and understandable. This finding supported the usability guideline to “Use headings that are unique from one another and conceptually related to the content they describe” [17].

Providing information alone was not appealing to these participants, and they reacted negatively to parts of the site that were too wordy or formal. This finding suggested that the content and features should focus on priority messages and tools rather than be exhaustive and overly detailed. Participants wanted both informational and interactive content that offered practical suggestions for improving nutrition, physical activity, and shopping and budgeting and that provided local community information. Providing practical information and information at a basic literacy level is consistent with research by the Children’s Partnership on the preferences of low-income and underserved populations [23].

### Round 3: Usability Testing

This round of testing provided feedback regarding the overall appropriateness, appeal, and ease of use of the draft website. Participants were generally very receptive to the website and its components. Users related strongly to nutrition and food budgeting areas, frequently disclosing personal stories related to their own nutritional and shopping practices. They continued to voice a strong interest in having content for children.

Overall, users stated that materials were easy to use and understand, even those with little or no computer experience. Participants provided suggestions to improve usability through simple formatting changes. They preferred the site to have limited text, larger font, and bulleted text to highlight key ideas. Participants wanted graphics to support content in order to improve understanding. Finally, some of the novice users requested adding user prompts and instructions to navigate links and menus on the pages. Overall, the participants’ recommendations directly support those provided by the 2006 US Department of Health and Human Services guidelines for improving health literacy [24].

With these usability test findings, the website needed only a few adjustments to the content and format. The website was then launched in the target communities within 6 weeks of completing the formative research process.

### Limitations

This paper presents an observation of attitudes, stated behaviors, preferences, and comments of the participating members of each group. Given our use of qualitative methods, statistical inference and generalization are not possible. Participants reflect a convenience sample of low-income mothers living in rural Maryland who volunteered for the study. Those who were most interested in issues related to nutrition, physical activity, or food budgeting, as well as those with computer/Internet skills may have been more likely to participate. Consequently, the study may be limited by self-selection bias.

Another limitation was the study’s focus on development of an Internet-based application rather than other technology-based applications that may be accessible to our target population. Taking into account the spectrum of access options now available may have enabled us to reach them in new and potentially more effective ways.

### Lessons Learned

As many researchers have found, recruiting rural populations can be difficult. Several different recruitment strategies were utilized in the current study. We found that recruitment through local community service providers was the most effective and efficient method. Recruitment was harder for the last two rounds, perhaps due to timing (ie, being conducted at the beginning and the end of the summer), weather, and minimal incentives.

We also attempted to over-recruit for each focus group. Although reminder calls were made, no-show rates and cancellations were high even among confirmed participants. A shift from scheduled focus groups to drop-in clinics to increase the flexibility of the time frame for participation did not improve participation rates.

Future data gathering efforts should use a variety of methods to promote participation, including varying locations of interview sites, offering varied incentive options, and enlisting the support of trusted service providers. Conducting intercept interviews in the local food stamp office or area frequented by the priority population may be an alternative strategy to test.

### Conclusions

This iterative formative research process illustrated the importance of participatory research. By the time we completed the third round of this research, we had greater confidence in our ability to meet the priority population’s needs and expectations because of their ongoing involvement. Participants had strong and clear opinions about what content to include and how to present it in order to make it easier to understand and access. Their recommendations were consistent with published guidelines on how to present materials to improve health literacy.

The research also highlighted the importance of adding new content and features on a regular basis as participants were not interested in a static site. Previous research has indicated that static sites may be a reason for drop-off in health website usage [16]. Keeping the website populated with new content would require continued formative research and usability testing, and we planned to conduct further usability testing after the initial implementation of the website.

This research has shown that despite barriers to technology use, low-income mothers were excited and interested in online materials designed for their needs. Addressing the needs of the “information and technology have-nots” is critical because they have the most to gain from access to appropriate materials. The challenge is to find out what works for our priority populations by moving from researcher-centric development to user-centered methods.

## Multimedia Appendix

Sample website designs used in Round 2
