# Supplementary figures and images for: Rural eHealth Nutrition Education for Limited-Income Families: An Iterative and User-Centered Design Approach
Source: J Med Internet Res. 2009 Jun 22;11(2):e21. doi: 10.2196/jmir.1148 (PMC2762801; doi:10.2196/jmir.1148)

## Slide 1
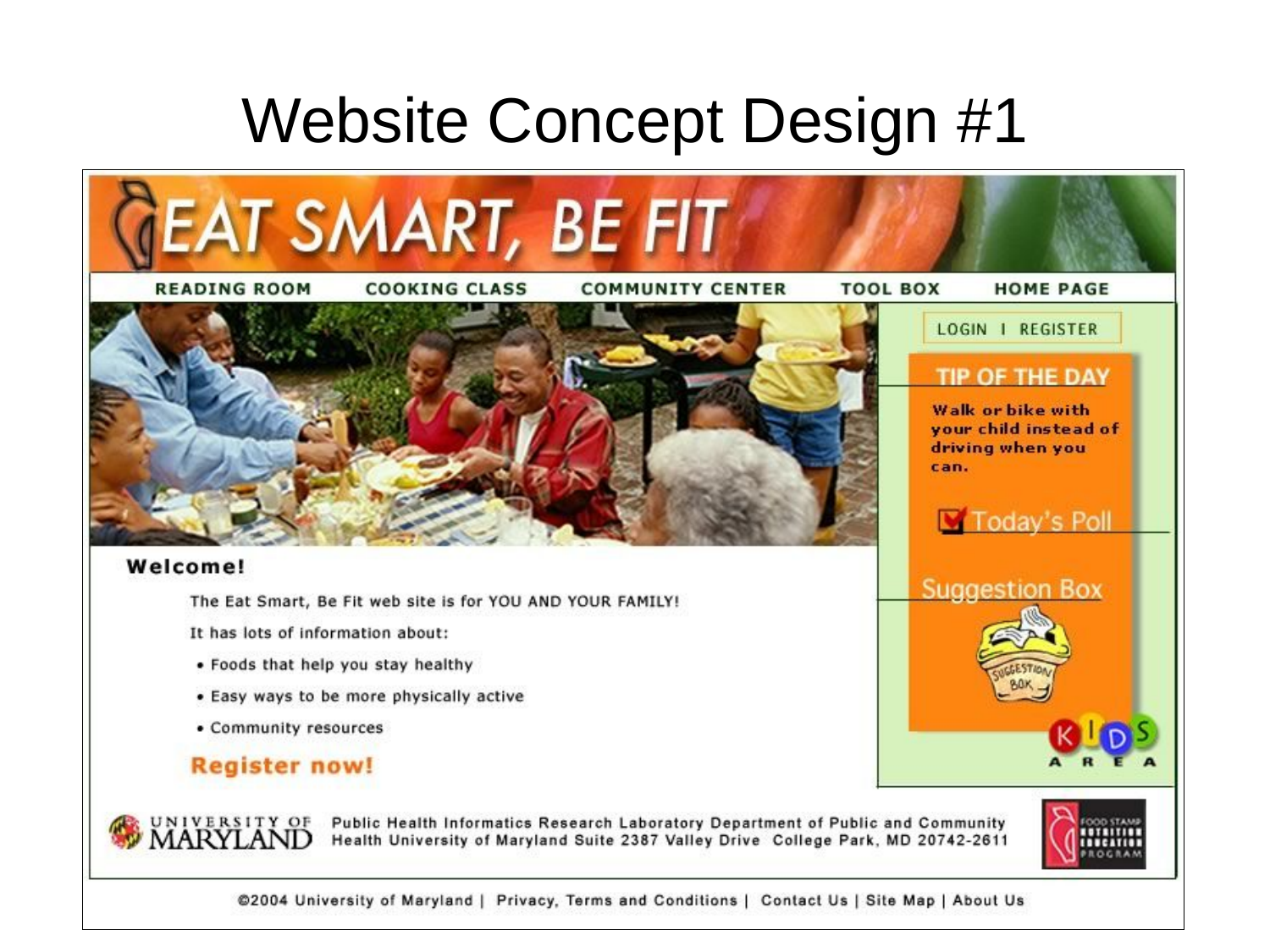

# Website Concept Design #1

## Slide 2
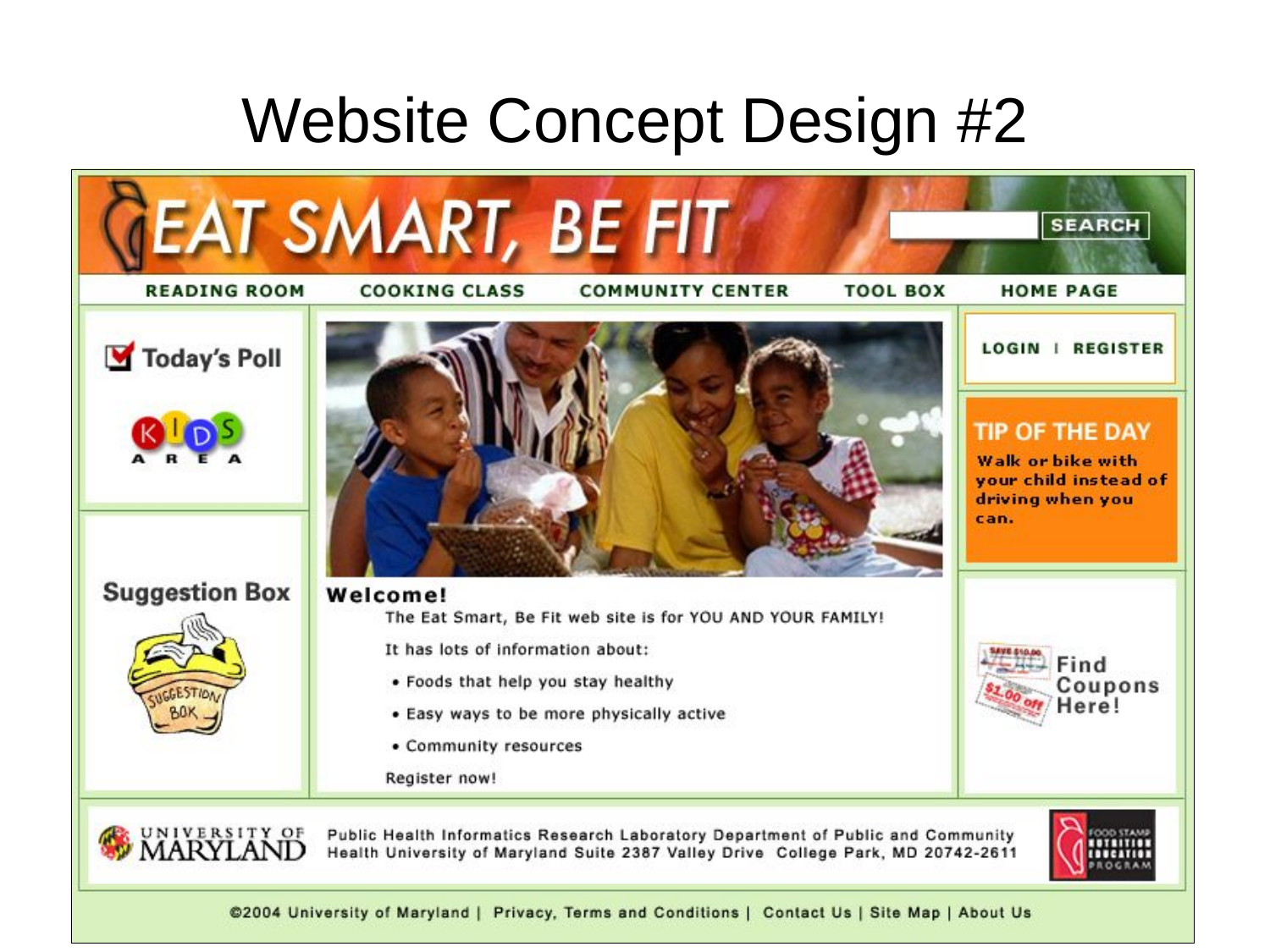

# Website Concept Design #2

## Slide 3
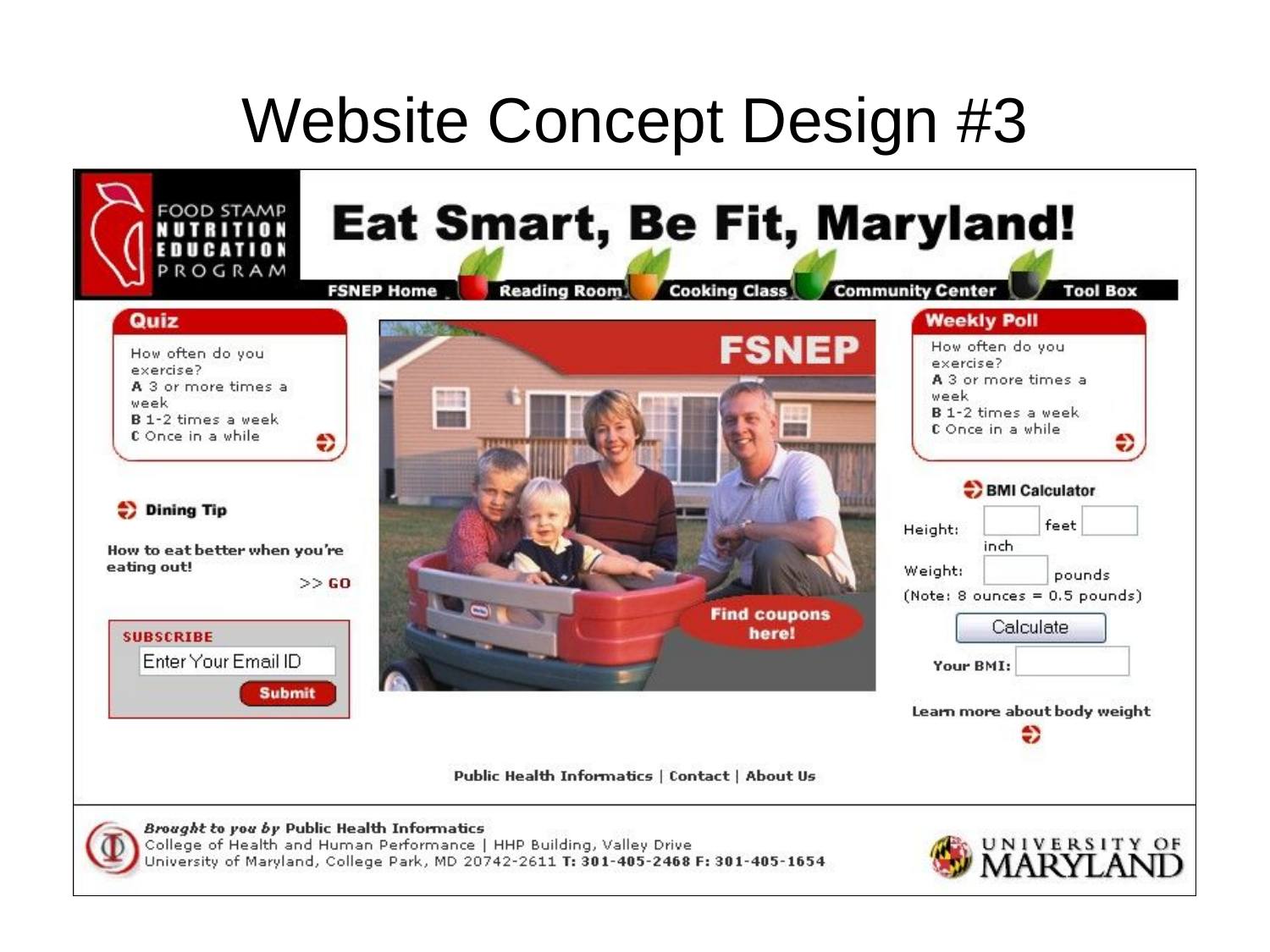

# Website Concept Design #3

Supplement: Supplementary file 1 [file jmir_v11i2e21_app1.ppt]
